# Valorification of Egyptian volcanic tuff as eco-sustainable blended cementitious materials

**DOI:** 10.1038/s41598-023-30612-0

**Published:** 2023-03-04

**Authors:** Khaled E. H. Eldahroty, A. A. Farghali, Nabila Shehata, O. A. Mohamed

**Affiliations:** 1 QC & Lab Manager, Minya Portland Cement Co, El Minya, Egypt; 2 Materials Science and Nanotechnology Department, Faculty of Postgraduate Studies for Advanced Sciences (PSAS), Beni-Suef University, Beni-Suef, 62511 Egypt; 3 Environmental Science and Industrial Development Department, Faculty of Postgraduate Studies for Advanced Sciences, Beni-Suef University, Beni-Suef, 62511 Egypt

**Keywords:** Engineering, Materials science

## Abstract

Rhyolite rocks extend from southern Egypt to northern Egypt in the Eastern Desert, and no effective economic exploitation of them has been discovered so far. The pozzolanic activities of different volcanic tuffs (VT) supplied from the Eastern Desert located in Egypt have been investigated as natural volcanic pozzolan materials to develop new green cementitious materials for achieving sustainability goals in the construction field. Experimentally in this paper, the pozzolanic activities of seven diverse specimens of Egyptian tuffs taken with standardized proportions of 75:25% (Cement: volcanic tuffs) were investigated. Pozzolanic features of such tuffs are examined comparatively via strength activity index (SAI), TGA, DTA, and the Frattini’s test. Chemical composition, petrographic, and XRD analysis were also performed for tuffs samples. The pozzolanic reaction degrees were determined according to the compressive strengths at 7, 28, 60 and 90 days with different replacement ratios (20, 25, 30 and 40%) of tuffs samples. Additionally, the micro-filler effects in mortar and concrete were determined by measuring the heat of hydration in mortar samples and the compressive strength of concrete with different additive ratios for tuffs samples besides, the concrete slump test. The results show that TF6 gives a lower cement heat of hydration value which is less than 270 J/g at 7 days. Also, its performance in concrete is better than silica fume at late strength (28 days) since the concrete index value is 106.2% by compared to the concrete index of silica fume 103.9 and therefore it can be used as an alternative to high price and quality variable silica fume (SF) for producing high-performance green concrete. Due to the good pozzolanic behavior proved by nearly most volcanic tuffs, along with their low cost, this study will be profitable for very auspicious the use of Egyptian volcanic tuffs for developing sustainable and eco‑friendly blended cement.

## Introduction

Cement production causes significant energy consumption, diminishes natural resources, and increases greenhouse gas emissions (mostly carbon dioxide). Thus, there is an urgent request to confront the environmental and economic challenges of the cement production industry to achieve sustainability aims^1–4^. Therefore, in the construction industry, the use of supplementary cementitious materials (SCMs) is an effective tool for achieving sustainability aims^5–8^.

The pozzolana is a siliceous and aluminous substance that has the ability to react with Ca(OH)_2_ at ordinary temperatures for forming relatively stable components that have cementitious properties, and is commonly used to ameliorate the physico-chemical and mechanical characteristics of concrete and cement pastes^9^.

Pozzolanic materials are utilized as SCMs with PC and are generally categorized according to their sources as natural or artificial substances. Natural pozzolanic materials can be available in the form of huge untapped deposits all over the world^10^.

Natural (volcanic origin) pozzolans are used as SCMs due to their advantages of increasing durability, lowering costs, reducing the heat of hydration, boosting freezing–thawing, concrete strength, and sulfate resistance, as well as environmental benefits^11–13^.Pyroclastic ash is the most famous volcanic ash utilized as a supplementary cementitious material^14^.

The influence of mineralogy on the characteristics of substances should be considered and also, and comparative studies among different natural pozzolans should be conducted to assess their usability of natural pozzolans. Besides, detecting of the hidden causes of the different reactivity of such natural pozzolans is also required^15^.

A little research has been done on the use of tuffs as a pozzolan in cement-based substances, but recently, the usage of tuff as a pozzolan has grown around the world. Further investigations are still required to enable the adequacy and efficient usage of natural pozzolans as SCMs^16–18^.

The utilities and features of tuff used as pozzolan in cementitious composite were investigated. The results confirmed that the tuffs were shown to be adequate as a supplementary binding substance in the cementitious composite as claimed by applicable standards where the tuff contains an abundant amount of SiO_2_ and Al_2_O_3_ in its chemical composition and abundant content of amorphous phase on rock texture. Thus, the degrees of pozzolanic reaction of the tuffs in the tuff-cement pastes gradually increased with the extension of treating time^16^.

The mixed mortar samples are workable paste (increasing in setting time) and the (VT) lies in the north, central, and south Egyptian eastern desert where the reserve of VT exceed two and half billion tons^19^.

The physico-mechanical characteristics of tuff taken from Korsimoro (Burkina Faso) were examined for employment as admixtures in the clinker for making pozzolanic cement. The findings confirmed that the Korsimoro tuff possesses pozzolanic activity and is proper for use as a substitutional binder material in the industry of cement^20^.

Volcanic tuff is considered one of the most natural pozzolanic substances which may be present in Middle East countries (e.g., Egypt, Saudi Arabia, and Jordan)^21^. Hence, it was economically selected for use as a SCMs due to its mutable mineralogical, chemical, and reactivity properties^22^.

The action of Jordanian volcanic tuff (VT) aggregates on the properties of cement mortar has been indicated. The findings revealed that the addition of Jordanian volcanic tuff in adequate proportion enhances these mortar properties^23^.Zeolite volcanic tuffs (taken from the Macicaş quarry that lies on the northwestern side of the Cluj-Napoca region) have been evaluated in the construction and building materials^24^.

The pozzolanic activities of diverse tuff specimens taken from northeast Turkey used as natural pozzolan on cement were studied. The results indicated that the rise in the ratio of silica (SO_2_) in the pozzolan boosts the pozzolanic activity^25^.

The investigation of the pozzolanic characteristics of the altered volcanic tuffs taken from Los Frailes caldera as pozzolans for the manufacturing of mortars was carried out using; chemical pozzolanicity tests (CPT) and electrical conductivity (ECT) after eight and fifteen days of curing. The findings of the electrical conductivity (ECT) and chemical pozzolanicity tests (CPT) analysis affirmed the pozzolanic characteristics of all tested specimens^26^.

The impact of partial substitution of cement by 0%, 5%, 10%, 15% and 20% of volcanic tuff on concrete's durability, workability, and mechanical characteristics has been evaluated. After 28 days of the age of concrete samples, the splitting tensile strength, flexural strength, thermal resistance, and ultrasonic pulse velocity was tested. Both absorption and Compressive strength experiments were performed at the 28 and 56- day ages of samples. The findings indicated that increasing the ratio of volcanic tuff substitution to cement at 28 days' age leads to reduce of flexural strength, compressive strength, slump, and splitting tensile strength. Moreover, the optimum value of the highest compressive strength (CS) of concrete was obtained at 56 days of age for samples when replacing the cement with 10% of the volcanic tuff^21^.

The use of partial substitution of OPC by 50% of volcanic tuffs in a Particular case enhanced the resistance of sulfate attack and concrete resistance to alkali-silica reactions^27^.

The pozzolanic activity of both rhyolite and trachyte has been studied, which has been partially replaced by the cement used in concrete production^12^. The results conducted that the ground trachyte can be used as pozzolana in the industry of cement; but, the ground rhyolite does not meet some of the limitations according to the relevant standards.

Hence, the main aim of this work was to investigate the pozzolanic performance of Egyptian tuffs as green cementitious materials in terms of strength, and durability.

## Empirical study

### Applicable materials

Portland cement shall be complying with (BS EN 197-1:2011) with strength limits CEMl 42.5N, and the alkali equivalent (EN 196-2 2005), not more than 0.6 used in this investigation. The rhyolite ignimbrites tuffs samples used in this research was collected from three different areas that covers rhyolite rocks of different ages and structures located in the eastern desert of Egypt (namely; north, central, and south of the eastern desert of Egypt); area no.1 is wadi abou hab from north of eastern desert which covered by 3 samples, area no.2 is wadi hamad in middle of the eastern desert which covered by 3 samples of rhyolite welded and non-welded ignimbrite and area no.3 is Wadi Ranga from south of eastern desert which covered by one sample of rhyolite. The order of the collection areas from oldest to newest was as follows; wadi Ranga, wadi abou hab and finally, wadi hamad.

### Experimental approaches

#### Collect and grind the tuffs

Seven tuffs samples used in this research was collected from three different areas located in Egypt's eastern desert (namely; north, central, and south of the eastern desert of Egypt) as follows; area no.1 is wadi abou hab from north of eastern desert which covered by 3 samples with codes TF1, TF2 and TF3. area no.2 is wadi hamad in middle of the eastern desert which is covered by 3 samples of rhyolite welded with code TF4 and non-welded ignimbrite with codes TF5 and TF6, and finally, area no.3 is Wadi Ranga from the south of eastern desert which covered by one sample of rhyolite with code TF7. Each sample is about composed sample consisting of three sub-samples each sample is about 5 kg and collected together in one sample, where each point is represented by the three samples that cover all variations expected in each area.

The samples are crushed, dried at 110 °C, and grinding in a lab ball mill and fineness is tested and measured according to BS 8615-1:2019 and EN 196-6:2005 standards of pozzolanic materials when used with PC (section 1: natural pozzolana and natural calcined pozzolana), the fineness of samples shall be expressed as proportions of mass that remained on the sieve 45 microns which determined by wet sieve according with the standard BS EN 451-2 or by dry alpine sieve (air jet ) in accordance with BS EN 933-10 and shall not exceed 40%, the samples are mixed and homogenized well by Shaker Mixer Turbula equipment. The results of the fineness test of the tuffs samples are listed in Table 1. Fixing the grinding time at 90 min in the laboratory ball in order to obtain high fineness, in addition to clarifying the difference between the samples when the grinding time is constant, to know the ease or difficulty of grinding for each type of rock. since the material is finer so it effects positively on compressive strength (filler effect).

**Table 1:** Sieve analysis of mixed volcanic tuffs samples with clinker and gypsum to prepare the test samples for physical and mechanical testes.

Sample notation	Residue % on 45 micron @ 90 min
TF`1	10
TF2	11
TF3	11.8
TF4	10.9
TF5	10
TF6	12
TF7	13.4

#### Chemical composition analysis

Table 2 displays the chemical textures of the substances used in this study via the X-Ray Fluorescence (XRF) technique.

**Table 2:** Chemical composition analysis of rock samples used in the investigation using XRF.

Chemical comp. (by mass in %)	TF1	TF2	TF3	TF4	TF5	TF6	TF7
Si_2_O	74.00	75.94	78.42	70.46	68.74	71.39	70.33
Al_2_O_3_	13.30	13.42	12.14	14.68	14.39	14.36	14.68
Fe_2_O_3_	4.90	0.84	0.92	2.58	2.90	2.36	2.74
CaO	1.13	0.68	0.68	1.18	1.41	1.03	1.39
MgO	0.16	0.65	0.49	0.61	0.72	0.35	0.65
P_2_O_5_	0.30	0.02	0.07	0.09	0.10	0.07	0.09
K_2_O	0.145	3.63	4.58	3.68	3.66	3.96	3.92
Na_2_O	0.001	2.40	1.93	5.34	5.16	5.16	4.99
SO_3_	0.20	0.04	0.04	0.06	0.19	0.01	0.33
Cl%	0.070	0.02	0.02	0.03	0.11	0.04	0.07
LOI	5.72	1.80	1.23	0.63	0.77	0.25	0.61
SUM	99.720	97.65	99.29	98.56	97.280	98.660	99.110
Na-eq	0.10	4.77	4.91	7.73	7.53	7.73	7.54
Insoluble Residue	70.7	79.50	84.40	75.56	65.06	67.66	64.1
Reactive Silica	3.04	–	–	–	6.33	2.67	9.90

#### Petrographic analysis

**Figure 1:**
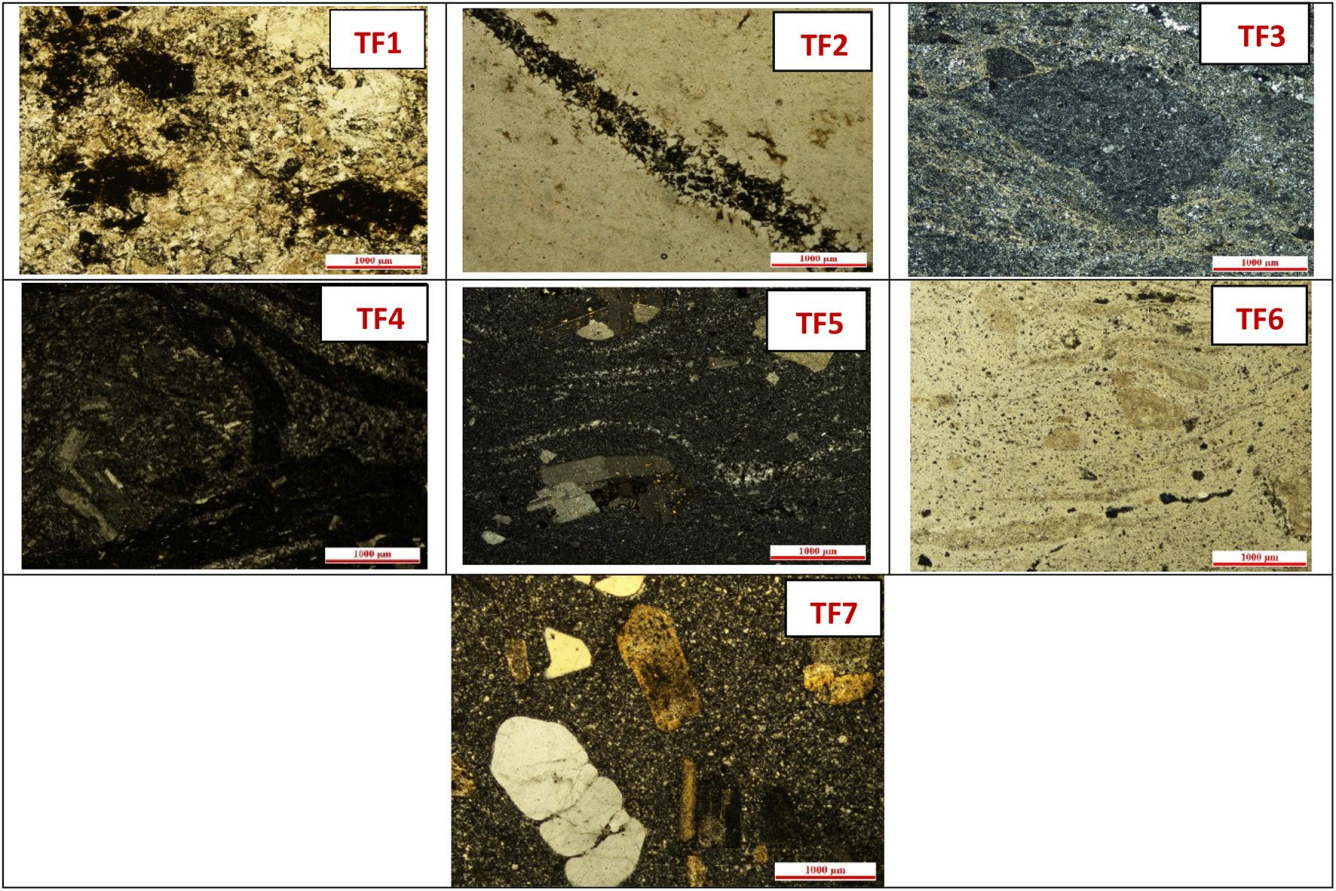
The petrographic description of the rhyolite ignimbrites tuffs samples.

By observation under the petrographic microscope, the thin divisions were prepared from the tuff samples. Based on the site of the original tuff, petrography characteristics mineralogical and were examined under a polarizing microscope using its thin divisions^16,28^ as shown in Fig. 1.

#### *X-ray diffraction (XRD) techniqu*e

**Figure 2:**
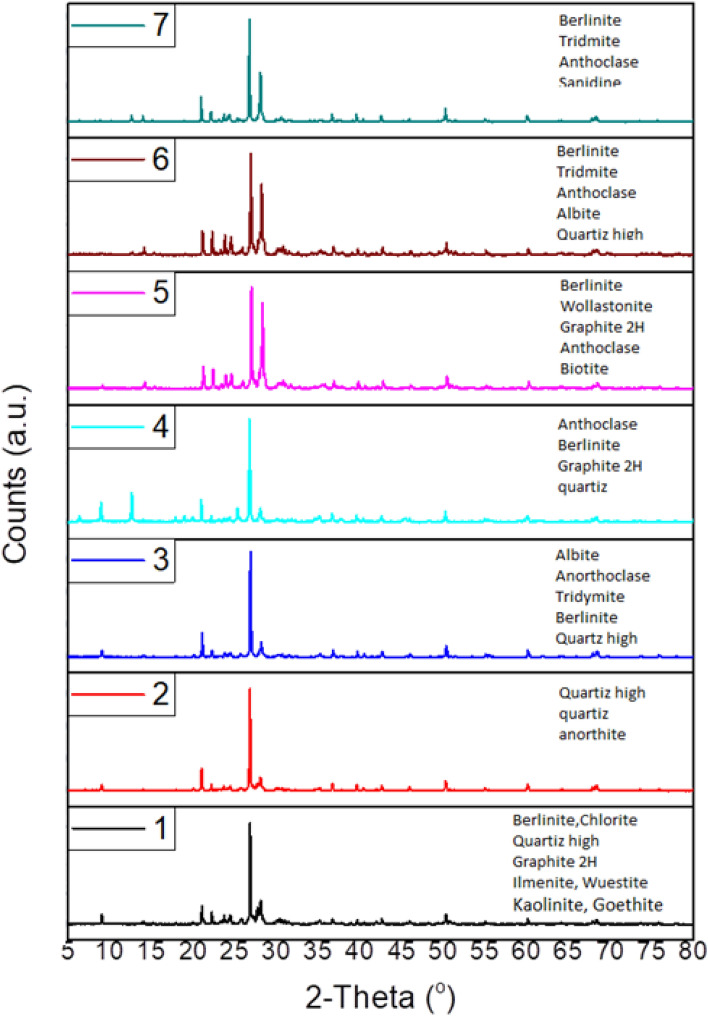
XRD spectra of the analyzed tuffs samples.

The mineral analysis of bulk powder samples was performed on a Phillips PW 340 X-ray diffractometer (40 kV and 30 mA using Cu Ka radiation). The results are shown in Fig. 2.

#### Determination of the pozzolanic activity

##### Preparation of mortars and pozzolanic activity test according to TS 25

In this test, the percentage of tuff substitutes was 25% of cement, weight per batch tested 112.5 gm of tuffs samples and 337.5gm of reference cement and mixed well using a tubular mixer for 20 min and CEN standard sand 1350 gm. The temperature should be 20 ± 2 °C and the humidity not be lower than 50%, the water used in preparing the specimens is distilled water, and all the previous components have the same laboratory temperature. By mixing according to EN 196-1 the specimen batch content is mixed and compacted in two layers by jolting apparatus and remove the mold to keep for 24 h in a humidity cabined of temperature 20 ± 1 °C and humidity not less than 90% and then de-molding the prisms and storage in the water tank to be ready for CS testing.

The behavior of pozzolana in the mortar was determined according to TS 25 for these seven specimens. In accordance with EN 196-1:2005 standard, the mortar specimens of the dimensions (40 mm × 40 mm × 160 mm) were prepared for testing the CS at 28 and 90 days. The mix batches were indicated in Table 3. The mortar specimens after molding were covered to inhibit any evaporation and then cured in a cabinet room at 23 ± 2 °C and relative humidity of more than 90% for 1 day. The CS of the specimens was tested at 28 and 90 days according to EN 196-1. BS EN 196-1 is equivalent to TS 25 standard. The setting characteristics of tuff samples are presented in Table 4.

**Table 3:** Amounts of materials required to prepare mixture batches for the determination of pozzolanic activity according to EN 196-1/2011 that comply to TS 25.

Contents	Control sample	Mix batch quantities
Cement (gm)	450	337.5
CEN sand (gm)	1350	1350
Ground sample (gm)	–	112.5
Water (ml)	225	225

**Table 4:** water of consistency and Setting time for Tuff samples according to EN 196-1/2011.

Sample name	Water consistency %	Setting time		Expansion (mm)
		Initial min	Final min	
Reference sample	27.5	170	200	1
TF1	29.2	215	255	0
TF2	28.3	195	215	0
TF3	27.9	175	205	1
TF4	28.8	160	200	1
TF5	28.4	170	205	0
TF6	27.6	180	215	0
TF7	27.7	200	240	0.5

##### The test of the strength activity index (SAI)

In such a case, the pozzolanic activity indexes of tuffs samples were determined in accordance with BS 8615-1:2019 standards. The Strength Activity Index is an important criterion for the performance of natural pozzolana in concrete tests and mortar tests. Therefore, the pozzolanic activity index was determined in Table 5 as the proportion of the average CS of the tested mortar sample to the average of the CS of the reference mortar sample.

**Table 5:** Compressive strength and pozzolanic active strength indexes of Tuff samples.

Sample	Tuff content, %	Compressive strength N/mm^2^		Pozzolanic active strength indexes (PASI), %	
		28 days	90 days	28 d ≥ 75	90 d ≥ 85
Reference mortar sample	0	52.60	65.80	100	100
TF1	25	43.7	52.7	83.1	80.1
TF2	25	39.0	49.0	74.1	74.5
TF3	25	37.4	49.5	71.1	75.2
TF4	25	35.7	48.3	67.9	73.4
TF5	25	41.6	51.9	79.1	78.9
TF6	25	43.8	53.9	83.3	81.9
TF7	25	44	54.3	83.7	82.5

##### The test of Frattini

**Figure 3:**
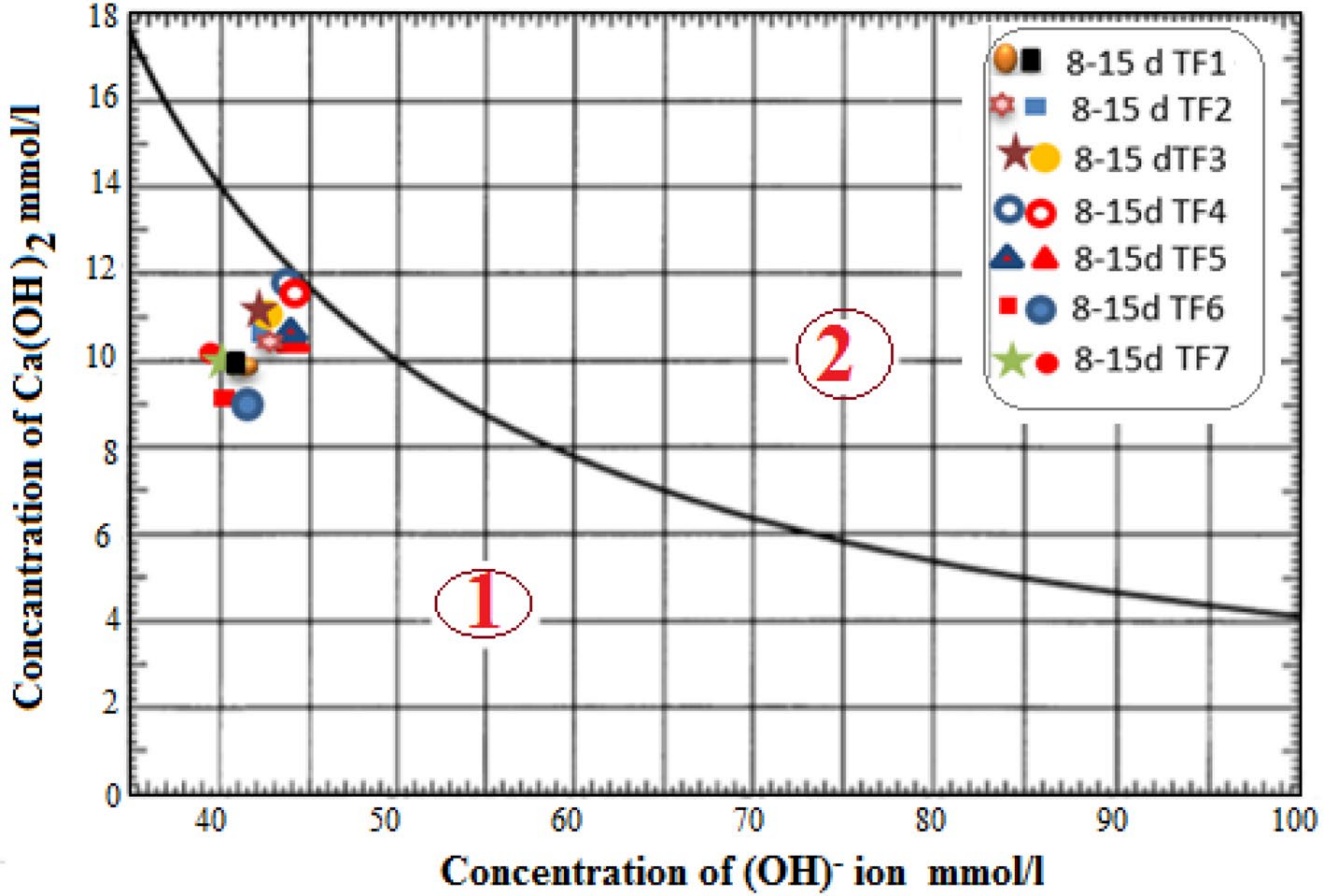
Variation of the pozzolanic features of Tuff samples at 8 and 15 days.

To decide the pozzolanic features in accordance with EN 196-5, the Frattini test was carried out by calculating the concentrations of OH^-^ and Na^+^ that contact with hydrated cement^12^. The results are shown in Fig. 3.

#### Pozzolanic reaction degrees and micro-filler effects

In accordance with EN 196-1, in this study, the mortar specimens were tested for CS at 7, 28, 60, and 90 days with different replacement percentages of mixed ratios (20, 25, 30, and 40%) of tuffs samples. The water content is fixed according to EN196-1 and (BS 8615-1/2019) in order to fix all the parameters to evaluate the substitution reaction.

The results of compressive strength are compared with the theoretical strength and filler effect in Table 6.

**Table 6:** Pozzolanic reaction degrees and micro-filler effects of tuff samples in the tuffs/ mortar.

Sample	Content of additive, %	Compressive strength N/mm^2^				Pozzolana Active Index (PAI) (%)			
		7 days	28 days	60 days	90 days	7 days	28 days	60 days	90 days
Reference mortar sample	0	36.00	52.60	64.00	65.80	100	100	100	100
Theoretical calculation	20	28.8	42.08	51.2	54.24	80	80	80	82.4
	25	27	39	48	49.35	75	75	75	75.0
	30	25.2	36.82	44.8	47.46	70	70	70	72.1
	40	21.6	31.56	38.4	40.68	60	60	60	61.8
TF1	20	27	47	52.6	53.4	75.0	89.4	82.2	81.2
	25	24.3	43.7	50.3	52.7	67.5	83.1	78.6	80.1
	30	21.2	31.4	44	44.1	58.9	59.7	68.8	67.0
	40	14.8	28.3	36.4	37.2	41.1	53.8	56.9	56.5
TF2	20	32.3	40.2	50.6	50.9	89.7	76.4	79.0	77.3
	25	23.0	39.0	45.0	49.0	63.9	74.1	70.3	74.5
	30	20.0	37.0	42.5	43.0	55.6	70.3	66.4	65.3
	40	17.0	25.7	37.7	39.7	47.2	48.9	58.9	60.3
TF3	20	26.3	41.2	51.8	52.04	73.1	78.3	80.9	79.1
	25	25	37.4	49.2	49.5	69.4	71.1	76.9	75.2
	30	21.9	33	38.5	39.6	60.8	62.7	60.2	60.2
	40	16.5	26.8	32.7	33.4	45.8	51.0	51.1	50.8
TF4	20	25.2	41.4	49.3	53.2	70.0	78.7	77.0	80.9
	25	21.7	35.7	47.6	48.3	60.3	67.9	74.4	73.4
	30	21	33.3	37	39.2	58.3	63.3	57.8	59.6
	40	16.7	27	31	32.4	46.4	51.3	48.4	49.2
TF5	20	32.8	45.1	54.5	60.1	91.1	85.7	85.2	91.3
	25	31.9	41.6	52.8	51.9	88.6	79.1	82.5	78.9
	30	28.9	39.3	40	42.8	80.3	74.7	62.5	65.0
	40	23.4	35.3	38	37	65.0	67.1	59.4	56.2
TF6	20	29.5	44.3	49.8	54.9	81.9	84.2	77.8	83.4
	25	28.6	43.8	48.2	53.9	79.4	83.3	75.3	81.9
	30	27.1	36.3	42.4	42.5	75.3	69.0	66.3	64.6
	40	23.6	31.6	40.4	36.4	65.6	60.1	63.1	55.3
TF7	20	28.8	46.1	53.1	55	80.0	87.6	83.0	83.6
	25	26.7	44	50.2	54.3	74.2	83.7	78.4	82.5
	30	25.3	36.2	43	44.7	70.3	68.8	67.2	67.9
	40	23.4	33.5	39.2	38.4	65.0	63.7	61.3	58.4

#### Heat of hydration tests

This test is performed by Semi- Adiabatic Calorimeter (the Semi-Adiabatic Langavant Method EN 196-9). The test mortar samples achieved according to EN 196-9 have a total mass of 1575 ± 1 g, consisting of the following mass proportions; 75% cement +25% samples (360.0 ± 0.5) g, CEN Standard sand (1080.0 ± 1) g, and deionized water (180.0 ± 0.5) g.

## Results

### Pozzolanic activities (PA) and their association with chemical compositions of tuffs

**Figure 4:**
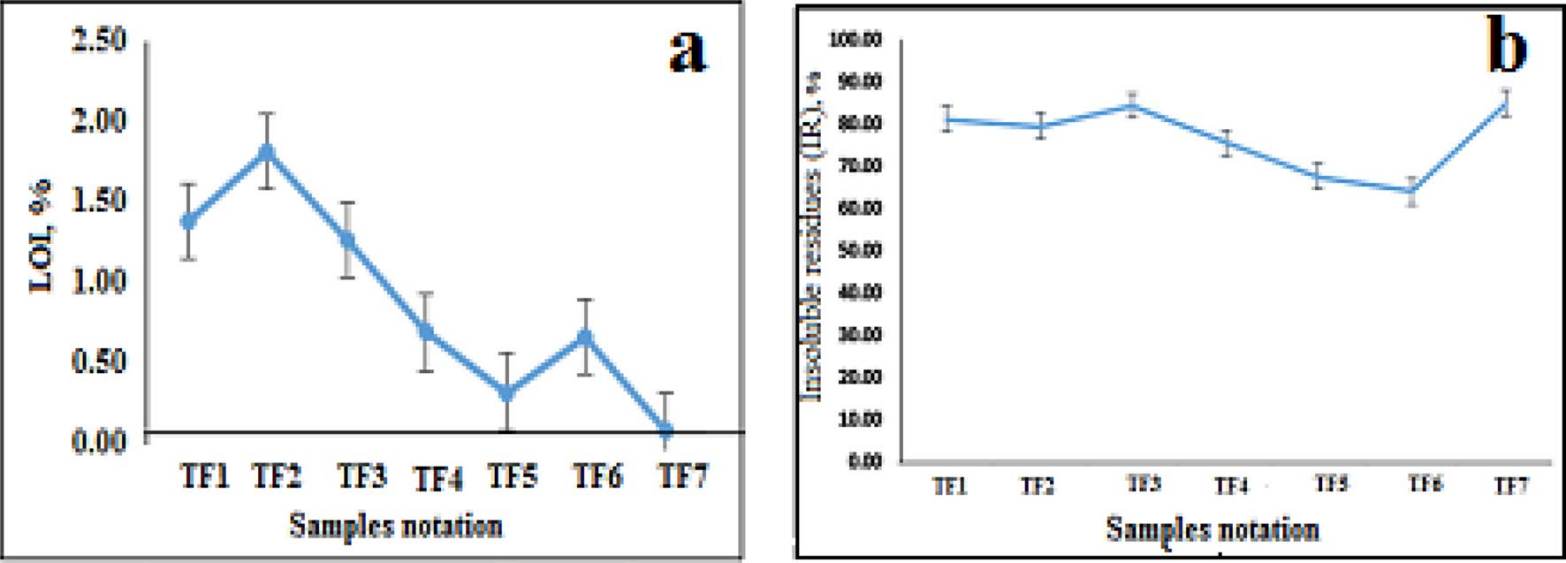
Loss on ignition (L.O.I.) in tuffs samples (**a**), and Insoluble residues (IR) values of tuffs samples (**b**).

As presented in Table 2, The chemical analysis results of collected tuffs samples are matched with BS 8615-1/2019 requirements. The requirements of BS 8615-1/2019 suggest that the total basic oxides should be ≥ 70%, i.e. SiO_2_ + Al_2_O_3_ + Fe_2_O_3_ ≥ 70%, and the findings indicate that the total basic oxides in tuffs samples are more than 70%. It should also be noted that in the requirements of BS 8615-1/2019, SO_3_ should be ≤ 3.0% and loss on ignition (L.O.I.) should be ≤ 7.0%. Thus, from the results of the chemical analysis offered in Table 2, it was assured that the samples comply with the requirements of BS 8615-1/2019 for SO_3_ content and loss on ignition (L.O.I.) in pozzolana standard, as in Fig. 4a. From major oxides analysis, it can be seen that all samples are in accordance with BS 8615-1/2019 requirements for chemical constituents which is the SiO_2_ content ˃ 45% with a decrease in MgO, SO_3_, and CaO.

As indicated in Table 2, the tuffs samples contain alkali as Na-eq which registers the smallest value of 0.1 in the TF1 sample whilst the value in TF2 and TF3 are equal to 4.77 and 4.91, respectively. Consequently, in accordance with BS 8615-1/2019 standards, these values of TF1 to TF3 comply with the pozzolana requirement. On the other hand, the highest values found in samples TF4, TF5, TF6 and TF7 are equal to 7.73, 7.53, 7.73 and 7.54, respectively which don't match with BS 8615-1/2019 where the maximum value for alkali as Na-eq should not more than 5.0%.

According to EN 196-2, the insoluble residues (IR) values have been calculated in Table 1 and Fig. 4b, which are in the range of 75.83–84.47% indicating a higher range as in samples of TF2, TF3, TF4 that reflect negatively on the value of reactive silica. As a rule, the higher the IR content, the lower would be the glass content and the reactive silica which results in lower development of the strength of cement. Thus, it can be noticed that the reactive silica contents are the highest values in samples TF1, TF5, TF6, and TF7 which equal 2.67–9.9%, respectively. But from the point of view that the reactive silica should be more than 25% according to BS 8615-1/2019^5^, there are no pozzolancity properties in tuffs samples but only slightly pozzolancity as shown in the results of the samples TF1, TF5, TF6, and TF7.

### Petrography

Figure 1 illustrates the petrographic description for the rhyolite ignimbrites tuffs samples^29^ as follows:

(I) Area no. 1: Ignimbrite rhyolite in wadi abou hab is formed mainly from acid volcanic rocks which it fine- grained, welded rhyolitic tuffs, and ill-sorted, this area is covered by 3 samples; TF1, TF2, and TF3. In sample TF1, the rock is fragmental and composed of feldspar crystals placed in a matrix of fine grains. Feldspar crystals are completely kaolinized. The matrix consists of feldspar, chlorite, and quartz. Opaque grains of iron oxides are common. In sample TF2 The rock is fine-grained, and composed of quartz and subordinate sericite. It is dissected by several veinlets of quartz or quartz and sericite. In sample TF3, the rock is fine- grained, composed of fine quartz and sericite and bigger rock fragments. It is dissected by numerous veinlets of quartz.
(II) Area no. 2: The rhyolitic ignimbrits of Wadi Hamad area are appeared as welded and non-welded varieties and represented by three samples TF4, TF5, and TF6. The welded ignimbrites of the sample (TF4); as can see in the thin section, these ignimbrites display welding and foliation, which ranges in width from a few millimeters to a centimeter. The rock fragments are commonly stretched or lenticular with tapered ends, forming fiamme. The thickness of fiamme varies significantly; consequently, the width- to- length ratio of these rock fragments varies considerably. These fiamme are aligned parallel to the bedding of the rock producing eutaxitic texture. The preferred orientation of such fiamme reflects compaction, either welding or diagenetic^30^. Some porphyritic fiamme contain spherulites, reflecting devitrification and suggesting that such fiamme most probably were vitrophyre. The mineral fragments are essentially represented by plagioclase. The plagioclase crystal fragments are subangular to subrounded and vary in size from ash to lapillus. The fiamme are largely porphyritic, with plagioclase as the dominant phenocryst phase, and are dacitic in composition. However, andesitic and basaltic angular to subrounded rock fragments are also present. Some porphyritic fiamme were likely vitrophyres as indicated by the presence of spherulites, which are formed by the devitrification of glassy materials. The ignimbrites of non-welded type of samples TF5 and TF6 as can be seen in thin section pictures are composed of rock and mineral fragments enclosed in the matrix, which varies in size from fine to very fine-grained. The rock fragments include essentially volcanic fragments and subordinate fiamme. The volcanic rock fragments are represented by porphyritic dacite and andesite. They vary in shape from subangular to rounded and range in size from coarse ash to lapillus. The fiamme are dacitic in composition and are commonly curved. They are commonly devitrified and occasionally contain phenocrysts of plagioclase, suggesting that some fiamme were vitrophyres. Fiamme are locally curved and wrap around the rock or mineral fragments. The plagioclase crystal fragments are subangular to subrounded and range in size from fine to coarse ash. The matrix is composed of fine fragments of plagioclase, quartz, and opaque minerals.
(III) Area no. 3: The Wadi Ranga volcanic rocks comprise porphyritic rhyolite and crystal tuffs, which belong to the Neoproterozoic island arc volcanic rocks of the Eastern Desert^31^. The petrographic **c**haracteristics of these rocks are given in the following paragraph:

Porphyritic rhyolite (TF7); The rock is a high porphyritic composed of plagioclase and quartz phenocrysts, collected in a fine-grained groundmass. Quartz phenocrysts or Plagioclase are locally gathered together forming a glomeroporphyritic texture. The plagioclase phenocrysts occur as big anhedral grains or subhedral to anhedral stout prisms. The plagioclase is highly converted to kaolinite or even fresh and locally extensively sericitized. Some phenocrysts show lamellar or simple twinning. Quartz is present as big and small anhedral grains, which locally display undulose extinction. Plagioclase phenocrysts and quartz are occasionally set via the groundmass constituents. In some samples, ferromagnesian minerals are represented by a few microphenocrysts of chlorite, which also present as fine scales in the groundmass and possibly formed at the expense of glass. The groundmass exhibits spherulitic texture, composed of intergrowths of quartz and plagioclase, reflecting rapid cooling. Chlorite (probably represents devitrified glass) occupies the interstices between the spherules. Opaques are present as small grains in the groundmass.

### Mineralogical results

XRD spectra of the tuffs samples are reported in Fig. 2. For mineralogical composition, all samples have a sum of minerals composition equal to 100% which confirms that the samples may do not or contain very little a glass phase.

### Physicomechanical aspects

#### Setting characteristics of tuffs samples

In accordance with EN 196-3, standard water of consistency and setting time were examined for prepared samples from 25% of ground materials (TF1–TF7) mixed with 75% of cement as shown in Table 4. The results indicate that the final and initial setting times of blended pastes ranged from 170 to 215 min and final setting times varied from 200 to 240 min, for water cement ratio ranging from 27.5 to 29.2%. The expansion test results for samples are low and it ranging from 0 to 1 mm. According to BS 8615-1:2019, the values were not exceeding twice of the cement reference sample and hence it complies with the pozzolanic required in the initial setting time and there is an increase in setting time more than the reference sample which is advanced in all blended samples except TF4 where it closed to the reference sample. However, the increase in water cement ratio in samples TF1, TF2, TF4 and TF5 is not advanced. Therefore, one of the differentiating factors in choosing pozzolana should be the low percentage of water needed to make a standard paste consistency. Another differentiating factor is the long setting time but not more than the standard limit, it reflects on the workability of concrete in a positive way by reducing the cost of concrete additions and also a positive reflection on the durability of concrete.

#### Pozzolanic activities

##### Strength activity index (SAI)

According to BS 8615-1:2019, as offered in Table 5, sample TF7 gives the highest value of 83.7% of the active index of compressive strength for 28 days of tested results whilst the TF4 sample represents the lowest value of 67.9%. All samples succeeded as pozzolana at 28 days of activity index, except for the TF4 sample, but in the case of the 90 days' active index test, the results were 73.4% in the TF4 sample and the highest value is 82.5% in the TF7 sample. With the required limit of 90 days, not all samples pass although cement grades 42.5N and 52.5N. The pozzolanic activity index was determined in Table 5 as the ratio of the CS of the tested sample to the CS of the reference sample. The results of CS showed the variance in the strength activity index (SAI) as selected samples (TF1, TF2, TF3, TF4, TF5, and TF6 & TF7) were used to investigate, evaluate, and correlate the variation in mineralogical content with the pozzolanic activity at intervals time. The values of the strength activity index for the investigated mortar samples at 28 and 90 days of curing ages are shown in Table 5. The mixture of samples (TF1, TF5, TF6 and TF7) showed that the SAI passes at 28 days but the TF4 sample doesn't pass as a pozzolanic material and all samples didn’t pass for SAI test at 90 days of curing ages. These findings reported that the pozzolanic activities of Tuff samples were corresponding with an increase in the active vitreous phases, which have a higher content of alumina and silica^32^.

##### Frattini test and pozzolanic reaction

Figure 3 shows the lime saturation curve of this test where the x-axis represents the (OH)^−^ ion concentration as the mmol/L, while the y-axis represents the concentration of (Ca)^+^ ion. This curve reflects the depletion of calcium hydroxide (CH) caused by cement hydration owing to the pozzolanic reaction and leads to a reduction in the concentration of (Ca)^+^ and (OH)^−^ where the reactive silica reacts with CH resulting from the hydration of OPC to generate the main hydration products as CSH gel that cause the long-term strength gain^33^. The results of pozzolanicity assessment indicated that the samples have pozzolanic characteristics in various degrees except for the TF4 sample which has non-pozzolanic properties. The Frattini test identification of the samples is followed in the order TF6 > TF7 > TF1 > TF5 > TF2 > TF3.

From the curve of the graph, the marked points representing samples of tuffs are merely falling in the lower and close to the lime saturation curve and thus are considered active in the pozzolanic property, or else they aren't inactive.

#### Thermal studies

**Figure 5:**
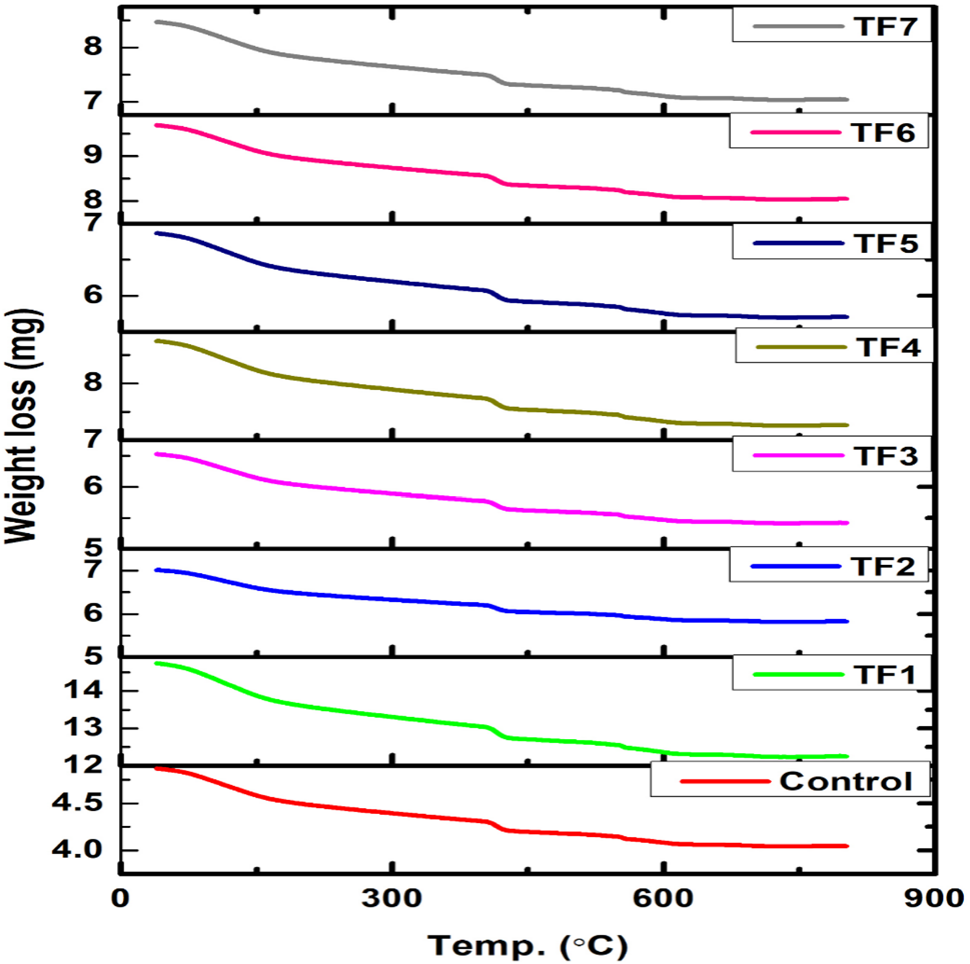
TGA analyses of Tuff samples at 60 days.

The cement paste is prepared for TGA test as grinding, mixed with acetone to fix the reaction of samples, and dried in a dryer at 110 °C. The findings of TGA tests of tuffs samples are indicated in Fig. 5. There are two main mass loss temperature ranges. The first mass loss is the removal of water from C–S–H gel which is between 100 and 180 °C, and the second is CH dehydrolaxation and it occurred between 450 and 520 °C^34–36^. The samples are dried with acetone and this process increases the CaCO_3_ between 600 and 750 °C (Taylor and Turner). The reaction of acetone in dried cement paste in an alkaline environment formed an organic substance and yielded irons of carbonates that were noticeable in the evaporated between 200 and 600 °C^37^. As can be seen from Fig. 5, the peak at 450–550 °C is due to the decomposition of portlandite where the smallest changes in CH dehydrolaxation were observed by TGA in samples TF5 and TF7 compared to the reference sample, due to possible pozzolanic activity^38^ followed by sample TF6 then sample TF1 (weaker reaction). Samples TF2, TF3, and TF4 are decayed at temperatures lower than those of portlandite, this is probably due to the presence of organic compounds and the peak of portlandite isn't much different from that of the reference sample implying little or no pozzolanic reaction, where sample TF4 show the smallest peak among them. Also, there is no significant effect of rock texture from welded and non-welded ignimbrite was observed on the pozzolancity. The portlandite peak does not differ much from that of the reference, which means little or no pozzolanic reaction. The smallest peak among them is TF4; in addition, the peak at 750–850 °C indicates the CaCO_3_.

### Pozzolanic reaction degrees and micro-filler effects

**Figure 6:**
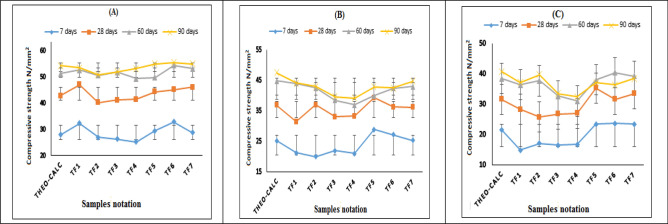
Compressive strength values at different substitution ratios for Tuff samples; (**A**) At substitution ratio of 20%, (**B**) At substitution ratio of 30% and (**C**) At substitution ratio of 40%.

As shown in Table 6, for the mixing ratio of 20%, the results indicated that the CS values of all samples ≥ the theoretical calculations of the strength at early strength (7 days), except for sample no. TF1, TF3, and TF4. At later strength (28 days), the samples TF1, TF5, TF6, and TF7 ˃ the theoretical calculation assumption, except samples TF2 and TF3 and TF4 are lower. At 60 days, samples TF1, TF3, TF5, and TF7 ˃ the theoretical calculation assumption, except samples TF2, TF4, and TF6 are lower. Finally, at 90 days, samples TF5, TF6, and TF7 ˃ the calculations of theoretical but TF1, TF2, TF3, and TF4 are lower, Fig. 6A. For the mixing ratio of 30%, the findings show that all samples at all late stages of strength are below the theoretical calculation assumption except for TF2 and TF5 are pass. But at the early stage of strength, samples TF5, TF6, and TF7 are passing Fig. 6B. In Fig. 6C, of mixing ratio of 40%, contrary to what is expected, the results showed that samples TF5, TF6, and TF7 are passed and this affirms that these samples have pozzolanic properties, and these findings comply with the results of chemical, and TGA investigation for samples.

As the results decline at a rate higher than the theoretical calculation of the effect of the amount of additions. This decline indicates the absence of the pozzolanic property and its presence in a small percentage of some tested samples. Where the results of compressive strength obtained from testing the samples in Table 6 and the theoretical calculation of compressive strength reduction due to the fillers effect are compared^39^. However, the extended difference in compressive strength is positive for samples containing large and small particle size of filler in mortars at lower replacement 20%, which means that there is a positive impact of filler on compressive strength up to 7, 28 and 60 days in case of filler particle size lower cement particle size (petrographic descriptions) this is clearly in results of compressive strength of high SAI in (TF1, TF6 and TF7) samples. TF2 is higher in 7 days compressive strength although it doesn't pass in SAI, it is related to filler effects as seen in The table of physical properties of ground of each volcanic tuff and cement, and is the highest fineness between all samples but the CS of substitutions decreasing at 28, 60 and 90 days late strength, from (petrographic analysis of thin section),the FT2 Tuff rock is fine-grained and composed of quartz and subordinate sericite, the sericite is alternate hydrothermal from feldspars minerals that it indicates no any little amorphous silica founded where PAI at 7 days for 20 and 25% substitutions are high values but decrease dramatically in late strength against the other samples.

### Impact of adding tuffs samples as a filler in mortar and concrete

#### Heat of hydration tests

**Figure 7:**
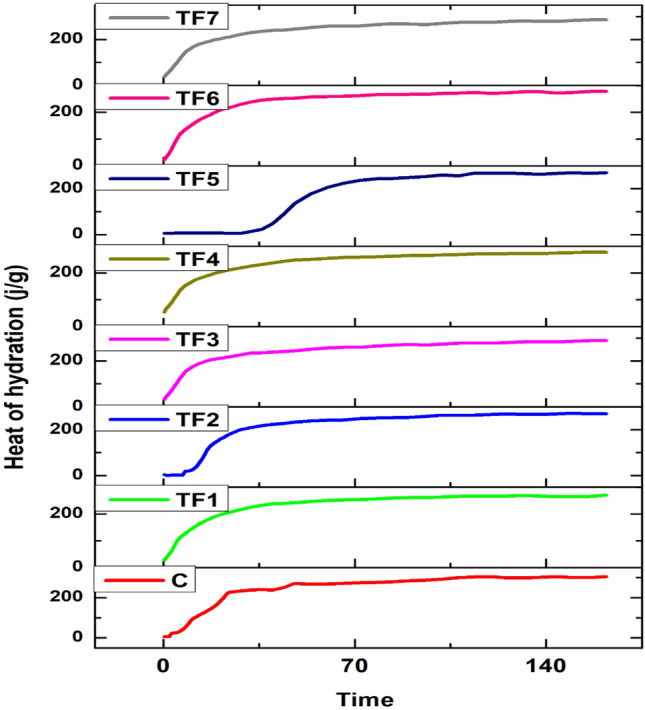
The graphic pattern of the effect of various tuffs on heat of hydration of mortar samples.

The graphic patterns of the accumulative heat generation due to the hydration reactions of cement were illustrated in Fig. 7. The results indicated that the heat of hydration of the cement reference sample after 7 days is 303.9 J/g and the depletion in the flow of cumulative heat of the blended study samples (TF1, TF2, TF3, TF4, TF5, TF6, and TF7) is greater than the reference sample and it occurs between 269.9 and 289.6 J/g, where the lack between the cement reference sample and the lowest one is 35 J/g while the difference between the studied samples' values during seven days is negligible. Subsequently, there is a positive impact on decreasing the accumulation of heat flow in the structure of concrete mass when replacing part of the cement with the studied tuffs samples^40^.

#### Filler effect in concrete

In this study, the CS of concrete with different additive ratios for tuffs samples was evaluated. All the aggregates and water- reduced concrete admixtures used in the trials from the plant materials, cement sample is OPC 42.5 N from Minya Portland cement company (Egypt). The water-reducing agent type G (R2008) is a superplasticizer and the latter improved the workability and concrete durability manufactured by CEMEX Egypt. In addition, 10% of silica fume was mixed with a reference sample to compare the pozzolanic and selected samples as a filler additive.

The SF from Grupo Ferro Atlantica–Spain. Moreover, some physical properties of SF according to ASTM C 1240–03 are given as follows; Specific Surface Area (21.5 cm^2^/g), Bulk Density (705 kg/m^3^), Fineness Retained on 45 µm (No. 325 Sieve) = 0.11, Autoclave Expansion (0.003 mm), Pozzolanic Activity Index = 117 and, 0.5% of Moisture Content. The concrete mix design consists of coarse of class 1 and 2, Fine aggregates of natural sand, and tap water all mixed in the following proportions in Table 7.

**Table 7:** Concrete mix design used in this investigation.

Materials	Mix weight, kg	Lab. trial weight, kg
OPC	400	12
Coarse aggregates 1	400	12
Coarse aggregates 2	670	20.1
Fine aggregates natural sand	760	22.8
Water	185	5.55
Admixture type G (R2008)	5.5	0.17

The tests of sand fine and coarse aggregates for grading size and specific gravity performed according to ASTM C33-03 (standard specification of concrete aggregates which defines the quality of the aggregate for grading, size, specific gravity, fine and coarse aggregates proportions and limits of deleterious substances in concrete raw materials.

The specific gravity of sand aggregates 1 and 2 is performed by defined container volume according to ASTM C29/C29M and the grading tested analysis according to ASTM C136-01 by using the representative sample from each content and by selecting, the sieves and the following results in tables verse the standard requirements as following of Table 8a–c. The physical properties of both volcanic tuff and cement were tabulated in Table 9.

**Table 8:** (a) Sieve analysis of coarse aggregate (Size 1), (b) Sieve analysis of coarse aggregate (Size 2), and (c) Sieve analysis of fine aggregate (Sand).

(a)					
Total weight = 3000 g					
Sieve size “mm”	Amount retained, g	% Retained	% Passing	ASTM C33 “Size # 7”	
				Min	Max
37.5	0	0.0	100.0	100	100
25	0	0.0	100.0	100	100
19	0	0.0	100.0	100	100
12.5	363	12.1	87.9	90	100
9.5	719	24.0	63.9	40	70
4.75	1898	63.3	0.7	0	15
2.36	17	0.6	0.1	0	5
1.18	0	0.0	0.1		
0.600	0	0.0	0.1		
Pan	3	0.1			
Size 1 aggregates specific gravity			2.65 kg/m^3^		

(b)					
Total weight = 3000 g					
Sieve size “mm”	Amount retained, g	% Retained	% Passing	ASTM C33 “Size # 56”	
				Min	Max
37.5	0	0.0	100.0	100	100
25	0	0.0	100.0	90	100
19	1146	38.2	61.8	40	85
12.5	1768	58.9	2.9	10	40
9.5	83	2.8	0.1	0	15
4.75	0	0.0	0.1	0	5
2.36	0	0.0	0.1		
1.18	0	0.0	0.1		
0.600	0	0.0	0.1		
Pan	3	0.1			
Size 2 aggregates specific gravity		2.7 kg/m^3^			

(c)					
Total weight = 594 g					
Sieve size “mm”	Amount retained, g	% Retained	% Passing	ASTM C33 LIMITS	
				Min	Max
9.5	0	0.0	**100.0**	100	100
4.75	3	0.5	**99.5**	95	100
2.36	11	1.9	**97.6**	80	100
1.18	63	10.6	**87.0**	50	85
0.600	183	30.8	**56.2**	25	60
0.300	200	33.7	**22.6**	5	30
0.150	104	17.5	**5.1**	0	10
0.075	7	1.2	**3.9**	0	3–5
Pan	23	3.9			
Sand specific gravity		2.6 kg/m^3^			

Significant values are in bold.

**Table 9:** Physical properties of ground of each of volcanic tuff and cement.

Samples/properties	TF1	TF2	TF3	TF4	TF5	TF6	TF7	Cement
Specific gravity g/cm^3^	2.6	2.65	2.57	2.68		2.71	2.7	3.14
Fineness residue % at 45 microns	10	8.6	11.8	10.9	10	12	13.4	10
Fineness residue % at 90 microns	0	0.3	2.3	1.4	1.7	1.2	3.1	0.5

### Concrete sample preparation and mix design

Prepare each concrete batch using a mini manual concrete mixer machine with a capacity of 180/5 L/cft with 16-RPM drum speed, where each batch consists of 12 kg cement (CEM1 42.5N, 12 kg coarse aggregates size 1 and 20.1 kg coarse aggregates size 2, and 22.8 kg, Fine aggregates natural sand, and 5.55 kg water and 0.17 kg Admixture type G (R2008), put the coarse aggregates size 1 and 2, sand and cement in concrete mixer and mix the drying for one minute and then add water with mixture during one minute and then left the mixer running for three minutes.

Stop the mixer and determine the initial slump test, return the concrete used in the mixer, and cover the mixer with a steel sheet for the next test of a slump in half an hour. After finishing the slump test, the mixtures of each cement tuffs mix were poured into moulds 15 cm × 15 cm × 15 cm and compacted by tapping rod cubes were de-moulded 24 ± 2 h and cured in drinking water. After the casting and storage in the curing water tank until the age of the test the compressive strength at 3, 7, and 28 days. The purpose of using blended cement with tuff in concrete trials in order to show the concrete rheology against OPC cement and also an example of mineral admixture (silica fume).

#### The compressive strength (CS)of concrete

The CS results at 3, 7 and 28 days are registered in Table 10. The aim of the active index calculations is to know the rate and effects of fillers and pozzolanicity against the silica fume (ASTM C1240) compare with cement reference sample. The samples TF3, and TF6 are used with different proportions of mixing with the cement reference sample and 10% silica fume (prepared according to ASTM C1240) mixed cement reference sample. The sample TF6 is selected to be subjected to mechanical activation by Grind it for two hours in the laboratory mill to get the remaining 5 microns' residue on 45 sieve in order to get close to the percentage of the fineness with silica fume, TF6 owing to its high pozzolanic properties compared to the rest of the samples based on the results of SAI, Frattini, and TGA tests.

**Table 10:** Pozzolanic activity indexes and compressive strengths of OPC, Tuff and SF samples in concrete.

Mixes	Compressive strength kg/cm^2^			Active index of mixed samples in concrete testes (%)		
	3 days	7 days	28 days	3 days	7 days	28 days
100% OPC	289	338.9	484.7	100	100	100
90% OPC + 10% TF3	272.7	310	433.7	94.30	91.40	89.50
85% OPC + 15% TF3	243.3	324.2	386.4	84.20	95.70	79.70
80% OPC + 20% TF3	226	285	382.8	78.20	84.10	78.90
75% OPC + 25% TF3	209.5	261.9	361.3	72.40	77.20	74.50
90% OPC + 10% TF6	283	351	515.4	97.90	103.60	106.20
75% OPC + 25% TF6	245	295	442	84.70	87	91.20
60% OPC + 40% TF6	212	250.2	379	73.30	73.80	78.20
90% OPC + 10% SF	335	363.1	503.8	115.90	107.10	103.90

From Table 10, the results indicated that the samples TF6 and TF3 of replacement 10% are close to in 3 and 7 days compressive strength. The TF3 sample of 20% replacement significantly reduces the early strength of concrete due to it does not possess any pozzolanic properties while the 10% replacement in the TF6 sample shows good CS values although when compared with silica fume 10%. The results were satisfactory for the sample TF6 and its performance in concrete is better than silica fume at late strength (28 days) since the concrete index value is 106.2% by compare with the concrete index of silica fume 103.9^41^ while silica fume shows good results in early strength (3 and 7 days) where the active index of silica fume are 115.9% and 107.1% respectively. The active indexes of TF6 at early strength (3 and 7 days) are 97.0% and 103.6%, respectively. This is attributed to the fact that TF6 is much coarser and is of lower pozzolancity than silica fume.

#### Concrete slump test

**Figure 8:**
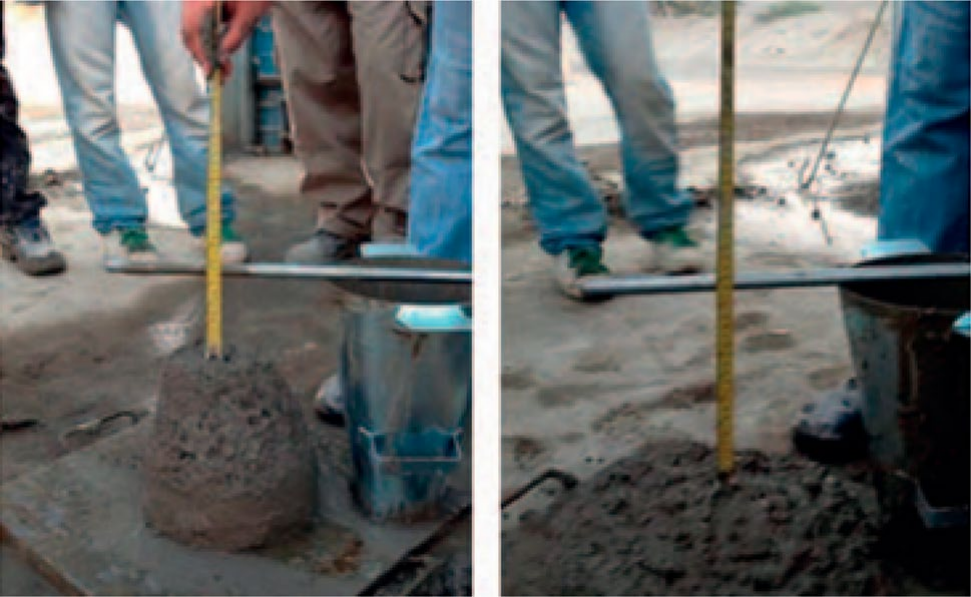
Photograph showing concrete slump test.

It was directly tested from fresh concrete preparation for the three proportions; OPC, 90% OPC + 10% TF6, and 90% OPC + 10% SF as seen in Fig. 8. The results indicated that the slump test of the cement reference sample at initial is 20 cm and after 30 min is 17 cm and after 60 min is 14 cm but by using 10% of sample TF6, the collapse occurred initially, at 30 min is 16 cm and at 60 min is 10 cm. When the later sample was compared with the 10%, the silica fume is 16 cm initially and 5 at 30 min, and 0 at 60 min. Thus, the workability of the sample TF6 is better than that of silica fume at 10% cement replacement. Where TF6 is Much less than SF in water demand, which is reflected positively in the slump value. it gives collapsed at an initial time followed by 16 cm at 30 min and 10 cm at 60 min if compared with SF slump values the SF gives 16 cm initial slump and 5 cm at 30 min and zero at 60 min hence rheology of the sample TF6 is better than SF. To improve the rheology of SF concrete, an increase in the concrete admixture of SF is required. Due to the color of TF6 being almost whitish, it consistently colors the concrete.

## Conclusions

The following summarizes the result of the analysis and discussion:

- Egyptian volcanic tuff can be used successfully as green cementitious material with 10 to 15% additions.
- The tuffs possess the substantial potential for pozzolanic activity as seen with the parameters of pozzolanic activity index, thus, tuffs due to their pozzolanic features can be used in the manufacturing of eco-sustainable blended cement.
- Nearly all tuffs samples have a long setting time, but not more than the standard limit, it is reflected in the workability of concrete positively in reducing the cost of concrete additions and also a positive reflection on the durability of concrete.
- For the micro-filler effects of tuffs samples at the lower replacement, 20% in the mortar, filler has a positive impact on compressive strength up to 7, 2, 8, and 60 days in case of the filler particle size is less than the cement particle size (petrographic descriptions). This positivity is clear in the results of the compressive strength of high SAI in (TF1, TF6, and TF7) samples.
- According to the results of the heat of hydration tests, there is a positive impact on decreasing the accumulation of heat flow in the structure of concrete mass when replacing part of the cement with the studied tuffs samples.
- TF6 sample can be used as an alternative to high price and quality variable silica fume (SF) for producing high-performance green concrete owing to its satisfactory result at late strength (28 days) and workability.

## Data Availability

All data generated or analyzed during this study are available upon request from all authors of this paper.
